# Glutamine-Induced Secretion of Intestinal Secretory Immunoglobulin A: A Mechanistic Perspective

**DOI:** 10.3389/fimmu.2016.00503

**Published:** 2016-11-24

**Authors:** Wenkai Ren, Kai Wang, Jie Yin, Shuai Chen, Gang Liu, Bie Tan, Guoyao Wu, Fuller W. Bazer, Yuanyi Peng, Yulong Yin

**Affiliations:** ^1^Key Laboratory of Agro-ecological Processes in Subtropical Region, Institute of Subtropical Agriculture, Chinese Academy of Sciences, Changsha, China; ^2^University of the Chinese Academy of Sciences, Beijing, China; ^3^Institute of Apicultural Research (IAR), Chinese Academy of Agricultural Sciences (CAAS), Beijing, China; ^4^Department of Animal Science, Texas A&M University, College Station, TX, USA; ^5^Chongqing Key Laboratory of Forage & Herbivore, College of Animal Science and Technology, Southwest University, Chongqing, China; ^6^College of Animal Science, South China Agricultural University, Guangzhou, China

**Keywords:** glutamine, intestinal microbiota, secretory IgA, T cells

## Abstract

Secretory immunoglobulin A (SIgA) is one important line of defense in the intestinal mucosal surface to protect the intestinal epithelium from enteric toxins and pathogenic microorganisms. Multiple factors, such as intestinal microbiota, intestinal cytokines, and nutrients are highly involved in production of SIgA in the intestine. Recently, glutamine has been shown to affect intestinal SIgA production; however, the underlying mechanism by which glutamine stimulates secretion of intestinal SIgA is unknown. Here, we review current knowledge regarding glutamine in intestinal immunity and show that glutamine-enhanced secretion of SIgA in the intestine may involve intestinal microbiota, intestinal antigen sampling and presentation, induction pathways for SIgA production by plasma cells (both T-dependent and T-independent pathway), and even transport of SIgA. Altogether, the glutamine-intestinal SIgA axis has broad therapeutic implications for intestinal SIgA-associated diseases, such as celiac disease, allergies, and inflammatory bowel disease.

## Introduction

The mammalian intestine is home to large numbers of bacteria, many of which invade the intestinal epithelium to enter the systemic circulation. In addition to bacteria, the intestine is challenged by viruses, parasites, food and environmental antigens, and bacterial metabolites. In order to maintain homeostasis, the intestinal mucosal surfaces have multiple layers of defense, including innate defenses and adaptive defenses. Innate defenses include mucus, antimicrobial substances (e.g., lysosome and defensins), and tight junctions (1). Secretory immunoglobulin A (SIgA) is the principal regulator of adaptive defenses on the intestinal mucosal surface of humans and many other mammals, such as mice, pigs, and rats. SIgA has critical roles in intestinal homeostasis by regulating immune responses *via* multiple mechanisms (2, 3). The characterized functions of SIgA in the intestine include: (1) immune exclusion *via* interacting with environmental antigens (e.g., bacteria, viruses, and toxins); (2) anti-inflammation by sampling intestinal antigens to induce Th2 or regulatory T cell-biased mucosal immune responses; (3) homeostasis of commensals by enhancing the cross talk between the probiotic bacteria and the intestinal mucosa (2, 3). Thus, the lack of SIgA in the intestine is associated with various intestinal diseases, such as necrotizing enterocolitis and gastrointestinal mucositis (3). Targets to increase secretion of intestinal SIgA are promising and directed at mitigating pathogenesis of diseases. Compelling evidence from well-designed investigations have shown that glutamine supplementation increases the abundance of SIgA in the intestine in various hosts, including rats (4, 5), mice (6, 7), Chinese Holstein calves (8), pigs (9), humans (10), and even broiler chickens (11). Similarly, we found that glutamine supplementation increases SIgA in the luminal contents of the jejunum and ileum, and the number of IgA^+^ plasma cells in the ileum in mice (12). However, underlying mechanisms by which glutamine promotes the production of intestinal SIgA are unknown. In our recent study, we found that dietary glutamine-mediated secretion of intestinal SIgA through effects on the intestinal microbiota, and T cell-dependent and T cell-independent pathways (12). In this review, we discuss the current evidence about underlying mechanisms whereby glutamine enhances production of intestinal SIgA.

## Generation of Intestinal SIgA

### M Cells and SIgA Production

Intestinal epithelia can be classified as villus epithelium (VE), which is mainly involved in digestion and absorption of nutrients, and follicle-associated epithelium (FAE), which promotes contact with luminal antigens to induce mucosal immune responses. VE contains primarily of enterocytes, scattered goblet cells, and, occasionally, enteroendocrine cells. Compared to the VE, FAE has fewer goblet cells, a thinner mucus layer, lack of expression of polymeric immunoglobulin receptor (pIgR) in enterocytes, and an absence of antimicrobial peptide-producing Paneth cells (13–15), which results in easier access of luminal antigens to FAE. Besides the above differences, FAE harbors a unique subset of epithelial cells, called microfold cells (M cells) (15, 16). M cells continuously sample and transport luminal antigens to the underlying gut-associated lymphoid tissue (GALT), where antigen-presenting cells (APCs), mainly immature DCs, capture the antigens and undergo maturation. After maturation, DCs migrate to the T-cell area of GALT to present antigens to T cells, which help in activation of antigen-specific B cells and ultimately production of sIgA by lamina propria IgA^+^ B cells (17).

The development of M cells in mice depends on the receptor activator of NF-κB ligand (RANKL) secreted by a subepithelial network of reticular cells and B cells. The binding of RANKL to its receptor, RANK (TNFRSF11a), promotes activation of the non-canonical (RelB) NF-κB signaling pathway, and expression of Spi-B that drives M cell fate determination and maturation (18, 19). Mice with *Tnfrsf11a* deletion lack intestinal M cells and have profound delays in emergence of lamina propria IgA^+^ plasma cells (20). The diminished amounts of fecal SIgA persist into adulthood, which suggests that antigen sampling by intestinal M cells is the principal pathway initiating mucosal SIgA production (20).

### Induction of Intestinal SIgA

For the induction of intestinal SIgA, both T cell-dependent and T cell-independent modes are proposed (3, 21–23). In the T cell-dependent model, M cells and intraepithelial dendritic cells (DCs) sample and deliver antigens from the intestinal lumen to APCs (like DCs and macrophages) in the underlying subepithelial dome region. Antigens are processed by APCs to peptide-derived antigens and then expressed with the major histocompatibility class II molecule (MHC-II). CD40 and the peptide-MHC-II complex on APCs bind to CD40L and T cell receptor (TCR) on T cells, respectively, to activate T cells in the interfollicular region. The activated T cells can promote B cell activation with signaling through the B cell receptor (BCR) and CD40 on B cells. Meanwhile, Th2 cytokines, such as transforming growth factor (TGF)-β1, interleukins (IL)-4, -5, -6, -10, and -13, are necessary for differentiation of immature B cells into IgA-secreting plasma cells. TGF-β1 is essential for activation and class switching recombination of IgM-positive B cells to IgA-positive B cells. Other Th2-derived ILs, including IL-4, -5, -6, -10, and -13, promote proliferation of IgA^+^ B cells and their differentiation into IgA-secreting plasma cells.

The production of most intestinal IgA in extrafollicular structures, such as isolated lymphoid follicles and lamina propria (LP), depends on the T cell-independent pathway. The B cells are activated by signaling through BCR and toll-like receptors (TLRs) recognizing microbial signatures. The release of the B cell-activating factor (BAFF), a member of the tumor necrosis factor family, a proliferation-inducing ligand (APRIL), the peptide hormone vasoactive intestinal peptide (VIP), IgA-inducing protein (IGIP), and nitric oxide (NO) from other cells (e.g., DCs), also promote T cell-independent mucosal IgA responses.

## Transportation of Intestinal SIgA

The process for transport of SIgA has also been well established (3, 22, 23). Briefly, most IgA-secreting plasma cells secrete IgA in the lamina propria as polymeric IgA (dimer or polymer), which is covalently linked to the joining (J) chain. The uptake of dIgA or pIgA is mediated by pIgR. pIgR is a 120 kDa transmembrane protein consisting of five extracellular immunoglobulin (Ig) homology domains, a transmembrane region and a cytoplasmic domain, and is expressed on the basolateral surface of epithelial cells. pIgR binds dIgA or pIgA at the basolateral side of epithelial cells, then the dIgA-pIgR or pIgA-pIgR complex is shuttled to the apical membrane of epithelial cells by vesicles. Upon reaching the apical side, pIgR is cleaved to release SIgA into the lumen of the intestine as a hybrid molecule including pIgA and secretory component (SC) from pIgR.

## Glutamine and Generation of Intestinal SIgA

Available evidence suggests that glutamine increases the abundance of intestinal SIgA, probably through the intestinal microbiota, induction pathway (T-dependent and T-independent), IgA-secreting plasma cells, and even transport of intestinal SIgA.

### Glutamine and Intestinal Microbiota

The first step in generation of SIgA from plasma cells is induction by intestinal antigens, mostly bacterial antigens from the lumen of the gut (24, 25). Germ-free mice have fewer IgA-expressing cells in the Peyer’s patches and lamina propria, and the colonization of germ-free mice with a microbiota quickly triggers production of IgA (26). Even a single strain of bacteria can effectively promote the secretion of intestinal SIgA. For example, not only *Streptococcus termophilus* (27), but most *Bifidobacterium*, such as *Bifidobacterium adolescentis BBMN23* (28), *Bifidobacterium longum BBMN68* (28), and *Bifidobacterium animalis* (29), induce the production of intestinal SIgA. However, some members of the microbiota (e.g., species of *Sutterella*) degrade both IgA and SC, thus they negatively influence the amount of intestinal SIgA (30). In a previous study, we found that glutamine modulates the intestinal microbial community in mice (31). At the phyla level, the content of Firmicutes in the jejunum and ileum of glutamine-supplemented mice is lower than for the control group, resulting in a shift in the Firmicutes-to-Bacteroidetes ratio to favor Bacteroidetes in the ileum (31). Meanwhile, glutamine supplementation increases the abundance of *Streptococcus* and *Bifidobacterium* in the jejunum, compared to the controls (31). As we discussed in a previous study (31), one possible mechanism is that glutamine supplementation changes the intestinal microenvironment, thereby altering the composition of intestinal microbiota (32). For instance, glutamine supplementation regulates utilization and metabolism of amino acids in bacteria in the small intestine in a niche-specific manner (33, 34), which may in turn affect the activity and number of certain microorganisms (31). Whether the decrease in Firmicutes-to-Bacteroidetes ratio promotes the production of intestinal SIgA is unknown, but we also found that arginine promotes the production of intestinal SIgA, coinciding with shifting the Firmicutes-to-Bacteroidetes ratio to favor Bacteroidetes in the jejunum and ileum (35). Indeed, monocolonization of the intestine of rats with *Bacteroides thetaiotaomicron* (belonging to Bacteroidetes) increases production of intestinal SIgA 6 days after colonization (36). Collectively, glutamine regulation of production of intestinal SIgA may be mediated by the intestinal microbiota. Indeed, our recent study using fluorescence *in situ* hybridization (FISH) analysis revealed that glutamine supplementation increases intestinal microbiota invasion into the wall of the ileum (12). Interestingly, disruption of the mouse intestinal microbiota with an antibiotic cocktail (37, 38) during glutamine supplementation abrogates the influence of glutamine supplementation on secretion of SIgA (12). Similarly, in antibiotic cocktail treated mice, dietary glutamine supplementation for 7 days fails to enhance intestinal SIgA production (12).

Paradoxically, it is widely known that glutamine decreases the translocation of bacteria from the gastrointestinal lumen to Peyer’s patches or mesenteric lymph nodes (MLNs) in rats (39, 40) and mice (7, 41). One possible reason for this conclusion is that it comes from the use of animal models with an impaired intestinal mucosal barrier (42, 43), thereby affecting the function of glutamine in intestinal bacteria. Indeed, although glutamine significantly decreases the translocation of bacteria across the gut in rats with chronic portal hypertension and common bile duct ligation, glutamine has little effect on bacterial translocation in rats subjected to a sham laparotomy (44). However, we found that dietary glutamine supplementation decreases bacterial translocation based on the lower bacterial load in the MLN of healthy mice (12). Another possible explanation is based on functions of SIgA to prevent the translocation of intestinal bacteria across the intestinal epithelium (2, 3, 22). Glutamine induces production of intestinal IgA, which inhibits the translocation of intestinal bacteria across the intestinal epithelium and reduces intestinal bacterial translocation after glutamine supplementation (40, 45). This finding is supported by evidence that glutamine decreases bacterial translocation in most models after a long period of usage (at least 8 days) (40, 41), while glutamine has little effect on bacterial translocation when supplemented for shorter periods of time (40, 46). It is possible that short periods of supplementation are insufficient to induce functional levels of SIgA. Thus, we propose the following model as to how glutamine supplementation promotes intestinal production of SIgA by influencing the intestinal microbiota. Glutamine supplementation affects the intestinal microbiota (31, 47) by increasing bacterial stimulation of the intestinal wall (12), which promotes intestinal secretion of SIgA, and the SIgA intercepts the invading bacteria and neutralizes them in the lamina propria (2, 3, 22, 48–50), which decreases the bacterial load in MLN after glutamine supplementation.

In conclusion, glutamine may modulate intestinal bacteria to effect production of intestinal SIgA and increases in SIgA inhibit the translocation of intestinal bacteria.

### Glutamine, Antigen Sampling, and Antigen Presentation

#### Glutamine and Mononuclear Phagocytes

Although M cells have critical roles in intestinal SIgA production, there are not publications that describe effects of glutamine on M cell maturation and function. This may be due to the scarcity of M cells available for research on their amino acid requirements and metabolism. Besides M cells, mononuclear phagocytes in VE can sample and deliver antigens from the intestinal lumen to APCs (51). LP contains CD11c^+^ mononuclear phagocytes: CD11c^hi^ CD103^+^ CD11b^+^ CX_3_CR1^−^ cells (CD103^+^ DCs) and CD11c^int^ CD103^−^ CD11b^+^ CX_3_CR1^+^ cells (CX_3_CR1^+^ macrophages), which capture antigen from the intestinal lumen by extending transepithelial dendrites (TEDs) from the LP into the lumen of the gut by penetrating tight junctions (52, 53). Unlike CX_3_CR1^+^ macrophages, CD103^+^ DCs migrate from the LP into the epithelium and crawl laterally while sending dendrites into the intestinal lumen to actively sample intestinal antigen (53). Indeed, activated B cells can move to the subepithelial dome of PPs, where they interact with DCs, which enhances IgA production by integrin αvβ8-mediated activation of TGF-β (54). The uptake of *Bacillus amyloliquefaciens* SQR9 by DCs induces the maturation and expression of CD80, CD86, CD40, MHCII, and cytokines in DCs, and secretion of SIgA (55). Also, lung CD103^+^ DCs and CD24^+^CD11b^+^ DCs have been shown to activate B cells through T cell-dependent or -independent pathways (56). Besides DCs, recent investigations have also shown that macrophages promote IgA production by B-1 cells in the intestine *via* TGF-β2-dependent manner (57, 58). Some amino acids affect migration and function of DCs. For example, DCs of mice with *Toxoplasma gondii* infection increase gamma-aminobutyric acid (GABA) secretion and exhibit a hyper-migratory phenotype because the increase in GABA activates GABAA receptor-mediated currents in *T. gondii*-infected DCs (59). Inhibition of GABA synthesis and signaling in *T. gondii*-infected DCs or blockade of GABAA receptor impairs function of DCs *in vitro*, including their transmigration capacity, motility, and chemotactic response to CCL19 (59). Glutamine increases the migration of *T. gondii*-infected bone marrow-derived DCs, while 2-(methylamino)-isobutyrate (MeAIB; inhibitor of glutamine transport by SNAT2), or methionine sulfoximine (MSO, a glutamine synthetase inhibitor) blocks glutamine-enhanced migration of *T. gondii*-infected bone marrow-derived DCs (60). Lower concentrations of glutamine diminish the function of monocyte-derived macrophages, such as cytokine synthesis, phagocytosis, and antigen presentation (61–63). Glutamine affects the expression of HLA-DR, intercellular adhesion molecule-1 (ICAM-1/CD54), Fc receptor for IgG (Fc gamma RI/CD64), complement receptors type 3 (CR3; CD11b/CD18) and type 4 (CR4; CD11c/CD18), and tetanus toxoid-induced antigen presentation on human monocyte-derived macrophages (61). Thus, glutamine may regulate intestinal SIgA production through its influence on intestinal antigen sampling and presentation by macrophages and DCs.

#### Glutamine, Epithelial Cells, and Goblet Cells

Villous epithelial cells expressing neonatal Fc receptor (FcRn) and goblet cells play a role in intestinal antigen sampling (51, 64). FcRn contributes to the uptake of intestinal antigens by VE cells because it functions as IgG secretion across the intestinal epithelium into the lumen and also IgG-dependent sampling of luminal antigens (65, 66). Although details of the process are unknown, goblet cells from the small intestine of mice deliver low molecular weight soluble antigens from the intestinal lumen to underlying CD103^+^ LP-DCs (64). The beneficial effects of glutamine on intestinal epithelial cells and goblet cells are well known (31, 67). For example, glutamine supplementation enhances expression of goblet cell-specific-mucin 4 in the mouse jejunum (31). However, it remains to be determined if glutamine affects intestinal antigen sampling by VE cells and goblet cells.

In conclusion, glutamine may influence sampling of intestinal antigens and presentation by APCs in intestine through M cells, macrophages, DCs, epithelial cells, and goblet cells.

### Glutamine and Th2 Lymphocytes

The activation of Th2 lymphocytes plays a critical role in the generation of intestinal SIgA by activating B cells (22, 23). Glutamine is known to affect the number and function of T lymphocytes, and their subgroups (helper T lymphocytes, cytotoxic T lymphocytes) in humans (68), mice (69), and rats (70). Unfortunately, there has been no further investigation into the effects of glutamine on subgroups of helper T lymphocytes, including Th1, Th2, Th17, and Tregs. However, in dextran sulfate sodium-induced colitis in mice, glutamine suppressed Th1/Th17 and expression of their associated cytokine expressions, but promoted Treg responses (71–73). Indeed, total parenteral nutrition decreases SIgA in the intestine and the abundance of Th2 cytokines, like IL-4 and IL-10, which are known to stimulate SIgA production *in vivo* (74). However, glutamine supplementation in such situations enhances expression of IL-4 and IL-10 and the abundance of SIgA in the intestine (74). We demonstrated that glutamine promotes Th2 responses in mice infected with bacteria or viruses (75, 76). In mice infected with porcine circovirus type 2 or *Pasteurella multocida*, glutamine supplementation increases expression of Th2 cytokines, like IL-6 and IL-10 (75, 76). However, others have reported that glutamine has little or even inhibitory effects on Th2 responses in some animal models (71, 77, 78). The discrepancy may be related to the animal model, dosage, route of administration, and/or duration of glutamine supplementation, as well as time of analyses and methodologies. We also found that the function of glutamine varies due to those variables (31, 75, 76, 78).

In our recent study, 7 days of dietary 1.0% glutamine supplementation had little effect on expression of IL-4 and IL-10 mRNAs in the ileum, but increased expression of IL-5, -6, and -13 mRNAs in the ileum (12). Meanwhile, glutamine supplementation increased TGF-β signaling based on greater expression of TGF-β1, -β2, and -β3 and TGF-β receptor 2 in the ileum of glutamine-supplemented mice, compared to control mice (12). Although dietary 1.0% glutamine supplementation for 7 days had little effect on the abundance of IL-5 protein in the ileum, glutamine supplementation enhances the abundance of IL-13 protein in the ileum (12). Glutamine supplementation also increased the abundance of TGF-β1 protein in the ileum (12). Interestingly, interference of IL-13 signaling during glutamine supplementation by intraperitoneal injection of the IL-13 antibody decreased expression of J-chain mRNA in the ileum (12). Collectively, glutamine promotes the secretion of SIgA in the intestine, and this may be mediated by Th2 cytokines, such as TGF-β and IL-13.

### Glutamine and T Cell-Independent Pathway

In LP, the production of most intestinal SIgA depends mainly on T cell-independent pathways associated with TLRs on B cells, and BAFF, APRIL, VIP, IGIP, and NO from other cells. Although effects of glutamine on expression of TLRs on B cells is not known, glutamine is an important energetic and biosynthetic nutrient for B lymphocytes (79) that may affect the expression of TLRs on B cells. In a mouse model with *P. multocida* infection, we found that glutamine supplementation affects the expression of TLRs (TLR-1 to TLR-9) in lung and spleen (76). In mice immunized with the inactivated *P. multocida* vaccine, glutamine supplementation increased the expression of TLR-6, -8, and -9 in spleen (78). In normal mice, we also found that glutamine supplementation affected expression of TLR-4 and -5 mRNAs in the ileum (31). These interesting results indicate that glutamine may affect the expression of TLRs on B cells, but direct evidence for that possibility is not available. There are few reports on the effect of glutamine on expression of BAFF, APRIL, VIP, and IGIP in innate immune cells. In our recent study, 1.0% glutamine supplementation increased expression of APRIL, BAFF, VIP receptor 1 and 2, and retinal dehydrogenases (RALDH 1 and 2) mRNAs in the ileum, but had little effect on the expression of inducible nitric oxide synthase (iNOS) mRNAs in the ileum (12). These compelling results suggest that glutamine may promote production of SIgA in the intestine *via* a T cell-independent pathway; however, more convincing evidence is needed to validate this hypothesis.

### Glutamine and IgA-Secreting Plasma Cells

Glutamine is an important energetic and biosynthetic nutrient for proliferation, survival, and function of B cells (68, 79, 80). Notably, glutamine significantly increases IgA-positive plasma cells in the jejunal LP in rats with proximal colonic resection (81). Although the septic rats with cecal ligation and puncture (CLP) have a lower number of intestinal LP IgA-positive plasma cells, compared with the sham CLP controls, parenteral glutamine supplementation increases the number of IgA-positive plasma cells in intestinal LP (82). Similarly, 7 days of 1.0% glutamine supplementation increased IgA-positive plasma cells in the ileum of mice compared with control mice without glutamine supplementation (12). The underlying mechanisms by which glutamine increases the number of the IgA-positive plasma cells are unknown. It is well known that retinoic acid released by DCs is involved to imprint gut-homing receptors, such as α4β7 integrin, CCR9, and CCR10 on IgA-positive B cells, resulting in the migration of IgA-positive B cells from Peyer’s patches to the LP (83, 84). However, it will be of interest to determine whether glutamine metabolism in DCs or in intestine also affects homing of IgA-positive B cells.

## Glutamine and Transport of Intestinal SIgA

The expression of pIgR is critical for transport of intestinal SIgA. Various intracellular signaling pathways are associated with the expression of pIgR, such as Janus kinase-signal transduction and activator of transcription (JAK-STAT), NF-κB, and mitogen-activated protein kinase (MAPK) (48, 85). Multiple cytokines produced by innate and adaptive immune cells in the host, including interferons (IFNs)-γ, IL-1, IL-4, IL-17, TNF, and lymphotoxin (LT)-β, are reported to regulate the expression of pIgR through intracellular signaling pathways (48, 85). For example, binding of IL-17 to its receptor activates the classical NF-κB pathway through MyD88-independent signaling, which results in nuclear translocation of an NF-κB dimer comprising p65/RelA and p50 subunits, and expression of pIgR because this NF-κB dimer may bind to a cognate element in intron 1 of the gene of pIgR (86). Indeed, inhibition of the classical NF-κB activation pathway by Bay11-7082 blocks the induction of pIgR expression by IL-17 in HT-29 cells (86). Mice deficient in the IL-17 receptor (*Il17r*^−/−^) have less SIgA in fecal content and lower expression of pIgR in both the small and large intestines, compared to wild-type mice (86). Similarly, intestinal microbes, especially segmented filamentous bacteria (SFB), which induce Th17 responses (87) and have a critical role in the production of intestinal SIgA (88) perhaps by affecting expression of pIgR. Indeed, intestinal microbiota can regulate the expression of pIgR (85). For example, in an *in vitro* study with HT-29 cells, the expression of pIgR was induced by co-culture with different strains of intestinal bacteria, such as *E. coli* and *Salmonella typhimurium* (89). An *in vivo* study with germ-free mice revealed that those mice have lower expression of pIgR, compared with mice with a normal microbiota. Further, monocolonization of commensal bacterium *B. thetaiotaomicron* to germ-free mice restored intestinal expression of pIgR to levels comparable to those in mice with a normal microbiota (90). The regulation of pIgR expression by intestinal microbiota may largely depend on the microbial products as most commensal bacteria are spatially segregated from the epithelial surface because of mucus, SIgA, and other antibacterial products. For example, some bacterial products, like butyrate and lipopolysaccharide, upregulate expression of pIgR (85, 89, 91, 92). Besides bacterial products, nutrients such as arginine (35) and retinoic acid (93, 94) can regulate the expression of pIgR. In our recent study, we found that glutamine supplementation affected expression of pIgR mRNA in our mouse model (12). Glutamine may affect pIgR expression through its effects on the intestinal microbiota, cytokines, and intracellular signaling pathways. The influence of glutamine on intestinal microbiota was discussed previously. Our research with various animal models revealed that glutamine influences production of multiple cytokines, such as IFN-γ (75), IL-1β (31, 76, 95), and IL-17 (31, 95). Furthermore, glutamine regulated the activation of intracellular signaling pathways, such as STAT, NF-κB, and MAPK (31, 95, 96). For example, glutamine affects the activation of NF-κB signaling by regulating the expression of NF-κB protein, the translocation of the dimer (p65 and p50) from the cytoplasm to nucleus, the degradation of p65 and IκB, and the expression of IκB kinase (31, 95, 96). Thus, glutamine may affect SIgA transport by affecting the expression of pIgR.

## Conclusion

**Figure 1:**
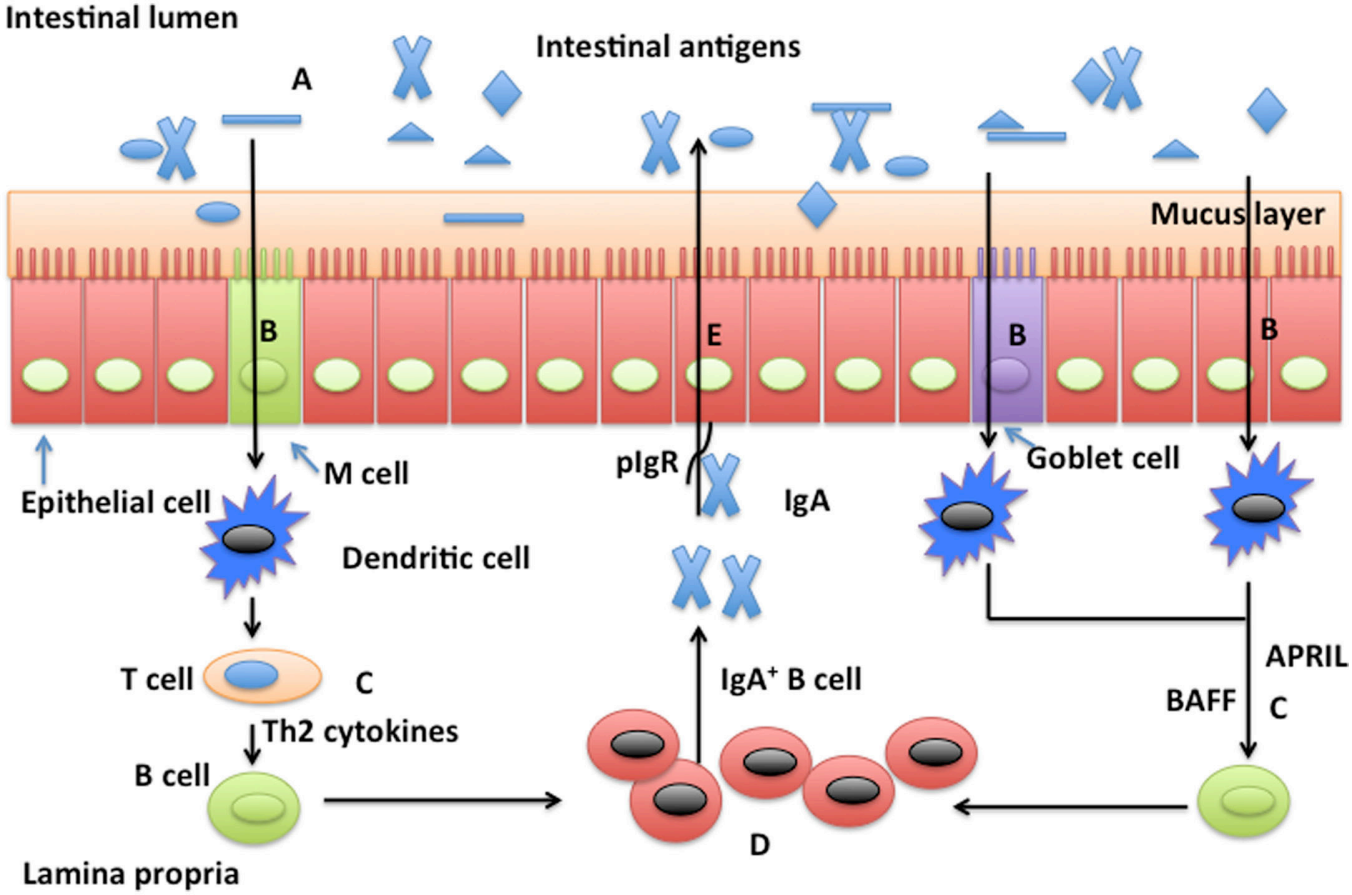
**Possible mechanisms whereby glutamine promotes secretion of intestinal secretory immunoglobulin A**. Intestinal secretory immunoglobulin A (SIgA) production requires stimulation *via* a T cell-dependent or a T cell-independent pathway. In the T cell-dependent pathway (left), M cells sample and deliver antigens from the intestinal lumen to dendritic cells (DCs) in the underlying subepitheilal dome region. DCs activate T cells in the interfollicular region and stimulate production of Th2 cytokines necessary for the differentiation of immature B cells into IgA-secreting plasma cells. In the T cell-independent pathway (right), release of the B cell-activating factor of the tumor necrosis factor family (BAFF) and a proliferation-inducing ligand (APRIL) from DCs promote T cell-independent mucosal IgA responses. Other factors, such as vasoactive intestinal peptide (VIP), IgA-inducing protein (IGIP), and nitric oxide (NO) also play important roles (not shown) in T-cell-independent pathways. SIgA is transported across the epithelium after binding to the polymeric immunoglobulin receptor (pIgR). In the intestinal lumen, SIgA binds intestinal antigens from microbes and diet. Glutamine may affect intestinal production of SIgA through intestinal microbiota **(A)**, antigen sampling and presentation **(B)**, induction pathways for SIgA production by plasma cells *via* either a T cell-dependent and T cell-independent pathway **(C)**, activation and homing of IgA^+^ plasma cells **(D)**, and transport of SIgA **(E)**.

It is well known that glutamine affects intestinal production of SIgA; however, the underlying mechanism by which glutamine promotes intestinal secretion of SIgA is unknown. The increase in knowledge of functions of glutamine in intestinal immunity suggests that glutamine affects intestinal production of SIgA through effects on intestinal microbiota, antigen sampling and presentation, induction pathways for SIgA production by plasma cells, including T-dependent and T-independent pathway, and even expression of pIgR (Figure 1). However, more well-designed experiments are required to provide convincing evidence to validate this hypothesis regarding relationships between glutamine and intestinal SIgA. New molecules affecting SIgA production are being found, such as Th17 cells (97) and innate lymphoid cells (98). It will be of interest to study the influence of glutamine on signaling by those cells. The amount of SIgA in the intestine has been associated with the pathogenesis of various intestinal diseases, such as inflammatory bowel disease (99, 100), food allergies (49, 101), and Celiac disease (102). Thus, manipulation of the glutamine-intestinal SIgA axis has a broad therapeutic potential for treating diseases associated with altered production of intestinal SIgA. As a functional amino acid, glutamine holds promise for improving the intestinal health of animals and humans (103–106).

## Author Contributions

WR, BT, and YY conceived this study. WR wrote the manuscript. KW, JY, SC, and GL provided critical discussion in manuscript preparation. FB, GW, and YP revised the manuscript.

## Conflict of Interest Statement

The authors declare that the research was conducted in the absence of any commercial or financial relationships that could be construed as a potential conflict of interest. The reviewer NS and handling Editor declared their shared affiliation, and the handling Editor states that the process nevertheless met the standards of a fair and objective review.

## Abbreviations

APCs, antigen-presenting cells; APRIL, a proliferation-inducing ligand; BAFF, B cell-activating factor of the tumor necrosis factor family; CLP, cecal ligation and puncture; DCs, dendritic cells; FAE, follicle-associated epithelium; FcRn, neonatal Fc receptor; FISH, fluorescence *in situ* hybridization; GABA, gamma-aminobutyric acid; GALT, gut-associated lymphoid tissue; IFN, interferon; IGIP, IgA-inducing protein; IL, interleukin; iNOS, inducible nitric oxide synthase; JAK, Janus kinase; LP, lamina propria; MAPK, mitogen-activated protein kinase; M cells, microfold cells; MHC-II, major histocompatibility class II molecule; MLN, mesenteric lymph nodes; NO, nitric oxide; pIgR, polymeric immunoglobulin receptor; RANKL, receptor activator of NF-κB ligand; SC, secretory component; SIgA, secretory IgA; STAT, signal transduction and activator of transcription; TEDs, extending transepithelial dendrites; TGF, transforming growth factor; TLRs, toll-like receptors; VE, villus epithelium; VIP, vasoactive intestinal peptide.

## References

[1] Heneghan AF Pierre JF Kudsk KA. JAK-STAT and intestinal mucosal immunology. JAKSTAT (2013) 2(4):e25530. 10.4161/jkst.25530 24416649 PMC3876429

[2] Corthesy B Multi-faceted functions of secretory IgA at mucosal surfaces. Front Immunol (2013) 4:185 10.3389/fimmu.2013.00185 23874333 PMC3709412

[3] Corthesy B Role of secretory IgA in infection and maintenance of homeostasis. Autoimmun Rev (2013) 12(6):661–5. 10.1016/j.autrev.2012.10.012 23201924

[4] Shu XL Yu TT Zhong JX Lei T. Effect of glutamine on intestinal barrier function following liver transplantation in rats. Eur Rev Med Pharmacol Sci (2014) 18(14):2058–64. 25027347

[5] Takechi H Mawatari K Harada N Nakaya Y Asakura M Aihara M Glutamine protects the small intestinal mucosa in anticancer drug-induced rat enteritis model. J Med Invest (2014) 61(1–2):59–64. 10.2152/jmi.61.59 24705750

[6] Fan J Meng Q Guo G Xie Y Xiu Y Li T Effects of enteral nutrition supplemented with glutamine on intestinal mucosal immunity in burned mice. Nutrition (2009) 25(2):233–9. 10.1016/j.nut.2008.08.009 18977117

[7] Santos RG Quirino IE Viana ML Generoso SV Nicoli JR Martins FS Effects of nitric oxide synthase inhibition on glutamine action in a bacterial translocation model. Br J Nutr (2014) 111(1):93–100. 10.1017/S0007114513001888 23773381

[8] Zhou Y Zhang P Deng G Liu X Lu D. Improvements of immune status, intestinal integrity and gain performance in the early-weaned calves parenterally supplemented with l-alanyl-l-glutamine dipeptide. Vet Immunol Immunopathol (2012) 145(1–2):134–42. 10.1016/j.vetimm.2011.10.020 22100191

[9] Zou XP Chen M Wei W Cao J Chen L Tian M. Effects of enteral immunonutrition on the maintenance of gut barrier function and immune function in pigs with severe acute pancreatitis. JPEN J Parenter Enteral Nutr (2010) 34(5):554–66. 10.1177/0148607110362691 20852186

[10] Fuentes-Orozco C Anaya-Prado R Gonzalez-Ojeda A Arenas-Marquez H Cabrera-Pivaral C Cervantes-Guevara G l-alanyl-l-glutamine-supplemented parenteral nutrition improves infectious morbidity in secondary peritonitis. Clin Nutr (2004) 23(1):13–21. 10.1016/S0261-5614(03)00055-4 14757388

[11] Bartell SM Batal AB. The effect of supplemental glutamine on growth performance, development of the gastrointestinal tract, and humoral immune response of broilers. Poult Sci (2007) 86(9):1940–7. 10.1093/ps/86.9.1940 17704382

[12] Wu M Xiao H Liu G Chen S Tan B Ren W Glutamine promotes intestinal SIgA secretion through intestinal microbiota and IL-13. Mol Nutr Food Res (2016) 60(7):1637–48. 10.1002/mnfr.201600026 27005687

[13] Pappo J Owen RL. Absence of secretory component expression by epithelial cells overlying rabbit gut-associated lymphoid tissue. Gastroenterology (1988) 95(5):1173–7. 10.1016/0016-5085(88)90347-2 2458985

[14] Giannasca PJ Giannasca KT Falk P Gordon JI Neutra MR. Regional differences in glycoconjugates of intestinal M cells in mice: potential targets for mucosal vaccines. Am J Physiol (1994) 267(6 Pt 1):G1108–21. 7810658 10.1152/ajpgi.1994.267.6.G1108

[15] Owen RL Uptake and transport of intestinal macromolecules and microorganisms by M cells in Peyer’s patches – a personal and historical perspective. Semin Immunol (1999) 11(3):157–63. 10.1006/smim.1999.0171 10381861

[16] Corr SC Gahan CC Hill C M-cells: origin, morphology and role in mucosal immunity and microbial pathogenesis. FEMS Immunol Med Microbiol (2008) 52(1):2–12. 10.1111/j.1574-695X.2007.00359.x 18081850

[17] Neutra MR Mantis NJ Kraehenbuhl JP. Collaboration of epithelial cells with organized mucosal lymphoid tissues. Nat Immunol (2001) 2(11):1004–9. 10.1038/ni1101-1004 11685223

[18] Kanaya T Hase K Takahashi D Fukuda S Hoshino K Sasaki I The Ets transcription factor Spi-B is essential for the differentiation of intestinal microfold cells. Nat Immunol (2012) 13(8):729–36. 10.1038/ni.2352 22706340 PMC3704196

[19] Tahoun A Mahajan S Paxton E Malterer G Donaldson DS Wang D *Salmonella* transforms follicle-associated epithelial cells into M cells to promote intestinal invasion. Cell Host Microbe (2012) 12(5):645–56. 10.1016/j.chom.2012.10.009 23159054

[20] Rios D Wood MB Li J Chassaing B Gewirtz AT Williams IR Antigen sampling by intestinal M cells is the principal pathway initiating mucosal IgA production to commensal enteric bacteria. Mucosal Immunol (2015) 9(4):907–16. 10.1038/mi.2015.121 26601902 PMC4917673

[21] Bemark M Boysen P Lycke NY. Induction of gut IgA production through T cell-dependent and T cell-independent pathways. Ann N Y Acad Sci (2012) 1247:97–116. 10.1111/j.1749-6632.2011.06378.x 22260403

[22] Pabst O. New concepts in the generation and functions of IgA. Nat Rev Immunol (2012) 12(12):821–32. 10.1038/nri3322 23103985

[23] Campos-Rodriguez R Godinez-Victoria M Abarca-Rojano E Pacheco-Yepez J Reyna-Garfias H Barbosa-Cabrera RE Stress modulates intestinal secretory immunoglobulin A. Front Integr Neurosci (2013) 7:86. 10.3389/fnint.2013.00086 24348350 PMC3845795

[24] Longman RS Yang Y Diehl GE Kim SV Littman DR. Microbiota: host interactions in mucosal homeostasis and systemic autoimmunity. Cold Spring Harb Symp Quant Biol (2013) 78:193–201. 10.1101/sqb.2013.78.020081 24913313 PMC4367195

[25] Mann ER Li X. Intestinal antigen-presenting cells in mucosal immune homeostasis: crosstalk between dendritic cells, macrophages and B-cells. World J Gastroenterol (2014) 20(29):9653–64. 10.3748/wjg.v20.i29.9653 25110405 PMC4123356

[26] Honda K Littman DR. The microbiota in adaptive immune homeostasis and disease. Nature (2016) 535(7610):75–84. 10.1038/nature18848 27383982

[27] Tormo Carnicer R Infante Pina D Rosello Mayans E Bartolome Comas R. [Intake of fermented milk containing *Lactobacillus casei* DN-114 001 and its effect on gut flora]. An Pediatr (Barc) (2006) 65(5):448–53. 10.1157/13094251 17184605

[28] Yang H Liu A Zhang M Ibrahim SA Pang Z Leng X Oral administration of live *Bifidobacterium* substrains isolated from centenarians enhances intestinal function in mice. Curr Microbiol (2009) 59(4):439–45. 10.1007/s00284-009-9457-0 19701668

[29] Martins FS Silva AA Vieira AT Barbosa FH Arantes RM Teixeira MM Comparative study of *Bifidobacterium* animalis, *Escherichia coli, Lactobacillus casei* and *Saccharomyces boulardii* probiotic properties. Arch Microbiol (2009) 191(8):623–30. 10.1007/s00203-009-0491-x 19526225

[30] Moon C Baldridge MT Wallace MA Burnham CA Virgin HW Stappenbeck TS. Vertically transmitted faecal IgA levels determine extra-chromosomal phenotypic variation. Nature (2015) 521(7550):90–3. 10.1038/nature14139 25686606 PMC4425643

[31] Ren W Duan J Yin J Liu G Cao Z Xiong X Dietary l-glutamine supplementation modulates microbial community and activates innate immunity in the mouse intestine. Amino Acids (2014) 46(10):2403–13. 10.1007/s00726-014-1793-0 25023447

[32] Dai ZL Wu G Zhu WY. Amino acid metabolism in intestinal bacteria: links between gut ecology and host health. Front Biosci (Landmark Ed) (2011) 16:1768–86. 10.2741/3820 21196263

[33] Dai ZL Li XL Xi PB Zhang J Wu G Zhu WY. l-Glutamine regulates amino acid utilization by intestinal bacteria. Amino Acids (2013) 45(3):501–12. 10.1007/s00726-012-1264-4 22451274

[34] Yang YX Dai ZL Zhu WY Important impacts of intestinal bacteria on utilization of dietary amino acids in pigs. Amino Acids (2014) 46(11):2489–501. 10.1007/s00726-014-1807-y 25063203

[35] Ren W Chen S Yin J Duan J Li T Liu G Dietary arginine supplementation of mice alters the microbial population and activates intestinal innate immunity. J Nutr (2014) 144(6):988–95. 10.3945/jn.114.192120 24670969

[36] Scharek L Hartmann L Heinevetter L Blaut M. Bifidobacterium adolescentis modulates the specific immune response to another human gut bacterium, *Bacteroides thetaiotaomicron*, in gnotobiotic rats. Immunobiology (2000) 202(5):429–41. 10.1016/S0171-2985(00)80102-3 11205373

[37] Suez J Korem T Zeevi D Zilberman-Schapira G Thaiss CA Maza O Artificial sweeteners induce glucose intolerance by altering the gut microbiota. Nature (2014) 514(7521):181–6. 10.1038/nature13793 25231862

[38] Vetizou M Pitt JM Daillere R Lepage P Waldschmitt N Flament C Anticancer immunotherapy by CTLA-4 blockade relies on the gut microbiota. Science (2015) 350(6264):1079–84. 10.1126/science.aad1329 26541610 PMC4721659

[39] Karatepe O Acet E Battal M Adas G Kemik A Altiok M Effects of glutamine and curcumin on bacterial translocation in jaundiced rats. World J Gastroenterol (2010) 16(34):4313–20. 10.3748/wjg.v16.i34.4313 20818815 PMC2937112

[40] Jiang JW Ren ZG Chen LY Jiang L Xie HY Zhou L Enteral supplementation with glycyl-glutamine improves intestinal barrier function after liver transplantation in rats. Hepatobiliary Pancreat Dis Int (2011) 10(4):380–5. 10.1016/S1499-3872(11)60064-7 21813386

[41] dos Santos R Viana ML Generoso SV Arantes RE Davisson Correia MI Cardoso VN. Glutamine supplementation decreases intestinal permeability and preserves gut mucosa integrity in an experimental mouse model. JPEN J Parenter Enteral Nutr (2010) 34(4):408–13. 10.1177/0148607110362530 20631386

[42] Anastasilakis CD Ioannidis O Gkiomisi AI Botsios D. Artificial nutrition and intestinal mucosal barrier functionality. Digestion (2013) 88(3):193–208. 10.1159/000353603 24247113

[43] Zhang X Jiang X Effects of enteral nutrition on the barrier function of the intestinal mucosa and dopamine receptor expression in rats with traumatic brain injury. JPEN J Parenter Enteral Nutr (2013) 39(1):114–23. 10.1177/0148607113501881 24047867

[44] Schimpl G Pesendorfer P Steinwender G Feierl G Ratschek M Hollwarth ME. Allopurinol and glutamine attenuate bacterial translocation in chronic portal hypertensive and common bile duct ligated growing rats. Gut (1996) 39(1):48–53. 10.1136/gut.39.1.48 8881808 PMC1383230

[45] Li Y Chen Y Zhang J Zhu JF Liu ZJ Liang SY Protective effect of glutamine-enriched early enteral nutrition on intestinal mucosal barrier injury after liver transplantation in rats. Am J Surg (2010) 199(1):35–42. 10.1016/j.amjsurg.2008.11.039 20103064

[46] Belmonte L Coeffier M Le Pessot F Miralles-Barrachina O Hiron M Leplingard A Effects of glutamine supplementation on gut barrier, glutathione content and acute phase response in malnourished rats during inflammatory shock. World J Gastroenterol (2007) 13(20):2833–40. 10.3748/wjg.v13.i20.2833 17569119 PMC4395635

[47] Sczesnak A Segata N Qin X Gevers D Petrosino JF Huttenhower C The genome of th17 cell-inducing segmented filamentous bacteria reveals extensive auxotrophy and adaptations to the intestinal environment. Cell Host Microbe (2011) 10(3):260–72. 10.1016/j.chom.2011.08.005 21925113 PMC3209701

[48] Johansen FE Kaetzel CS. Regulation of the polymeric immunoglobulin receptor and IgA transport: new advances in environmental factors that stimulate pIgR expression and its role in mucosal immunity. Mucosal Immunol (2011) 4(6):598–602. 10.1038/mi.2011.37 21956244 PMC3196803

[49] Mantis NJ Rol N Corthesy B. Secretory IgA’s complex roles in immunity and mucosal homeostasis in the gut. Mucosal Immunol (2011) 4(6):603–11. 10.1038/mi.2011.41 21975936 PMC3774538

[50] Geuking MB McCoy KD Macpherson AJ. The function of secretory IgA in the context of the intestinal continuum of adaptive immune responses in host-microbial mutualism. Semin Immunol (2012) 24(1):36–42. 10.1016/j.smim.2011.11.005 22138187

[51] Schulz O Pabst O. Antigen sampling in the small intestine. Trends Immunol (2013) 34(4):155–61. 10.1016/j.it.2012.09.006 23083727

[52] Varol C Vallon-Eberhard A Elinav E Aychek T Shapira Y Luche H Intestinal lamina propria dendritic cell subsets have different origin and functions. Immunity (2009) 31(3):502–12. 10.1016/j.immuni.2009.06.025 19733097

[53] Farache J Koren I Milo I Gurevich I Kim KW Zigmond E Luminal bacteria recruit CD103+ dendritic cells into the intestinal epithelium to sample bacterial antigens for presentation. Immunity (2013) 38(3):581–95. 10.1016/j.immuni.2013.01.009 23395676 PMC4115273

[54] Reboldi A Arnon TI Rodda LB Atakilit A Sheppard D Cyster JG. IgA production requires B cell interaction with subepithelial dendritic cells in Peyer’s patches. Science (2016) 352(6287):aaf4822. 10.1126/science.aaf4822 27174992 PMC4890166

[55] Huang L Qin T Yin Y Gao X Lin J Yang Q Bacillus amyloliquefaciens SQR9 induces dendritic cell maturation and enhances the immune response against inactivated avian influenza virus. Sci Rep (2016) 6:21363. 10.1038/srep21363 26892720 PMC4759567

[56] Ruane D Chorny A Lee H Faith J Pandey G Shan M Microbiota regulate the ability of lung dendritic cells to induce IgA class-switch recombination and generate protective gastrointestinal immune responses. J Exp Med (2016) 213(1):53–73. 10.1084/jem.20150567 26712806 PMC4710201

[57] Okabe Y Medzhitov R. Tissue-specific signals control reversible program of localization and functional polarization of macrophages. Cell (2014) 157(4):832–44. 10.1016/j.cell.2014.04.016 24792964 PMC4137874

[58] Cassado Ados A D’Imperio Lima MR Bortoluci KR. Revisiting mouse peritoneal macrophages: heterogeneity, development, and function. Front Immunol (2015) 6:225. 10.3389/fimmu.2015.00225 26042120 PMC4437037

[59] Fuks JM Arrighi RB Weidner JM Kumar Mendu S Jin Z Wallin RP GABAergic signaling is linked to a hypermigratory phenotype in dendritic cells infected by *Toxoplasma gondii*. PLoS Pathog (2012) 8(12):e1003051. 10.1371/journal.ppat.1003051 23236276 PMC3516538

[60] Lee IP Evans AK Yang C Works MG Kumar V De Miguel Z *Toxoplasma gondii* is dependent on glutamine and alters migratory profile of infected host bone marrow derived immune cells through SNAT2 and CXCR4 pathways. PLoS One (2014) 9(10):e109803. 10.1371/journal.pone.0109803 25299045 PMC4192591

[61] Spittler A Winkler S Gotzinger P Oehler R Willheim M Tempfer C Influence of glutamine on the phenotype and function of human monocytes. Blood (1995) 86(4):1564–9. 7632965

[62] Rogero MM Tirapegui J Vinolo MA Borges MC de Castro IA Pires IS Dietary glutamine supplementation increases the activity of peritoneal macrophages and hemopoiesis in early-weaned mice inoculated with *Mycobacterium bovis* bacillus Calmette-Guerin. J Nutr (2008) 138(7):1343–8. 18567758 10.1093/jn/138.7.1343

[63] Xiao W Chen P Dong J Wang R Luo B. Dietary glutamine supplementation partly reverses impaired macrophage function resulting from overload training in rats. Int J Sport Nutr Exerc Metab (2015) 25(2):179–87. 10.1123/ijsnem.2014-0118 25028814

[64] McDole JR Wheeler LW McDonald KG Wang B Konjufca V Knoop KA Goblet cells deliver luminal antigen to CD103+ dendritic cells in the small intestine. Nature (2012) 483(7389):345–9. 10.1038/nature10863 22422267 PMC3313460

[65] Yoshida M Claypool SM Wagner JS Mizoguchi E Mizoguchi A Roopenian DC Human neonatal Fc receptor mediates transport of IgG into luminal secretions for delivery of antigens to mucosal dendritic cells. Immunity (2004) 20(6):769–83. 10.1016/j.immuni.2004.05.007 15189741

[66] Yoshida M Kobayashi K Kuo TT Bry L Glickman JN Claypool SM Neonatal Fc receptor for IgG regulates mucosal immune responses to luminal bacteria. J Clin Invest (2006) 116(8):2142–51. 10.1172/JCI27821 16841095 PMC1501111

[67] Marc Rhoads J Wu G. Glutamine, arginine, and leucine signaling in the intestine. Amino Acids (2009) 37(1):111–22. 10.1007/s00726-008-0225-4 19130170

[68] Cetinbas F Yelken B Gulbas Z. Role of glutamine administration on cellular immunity after total parenteral nutrition enriched with glutamine in patients with systemic inflammatory response syndrome. J Crit Care (2010) 25(4):e661–6. 10.1016/j.jcrc.2010.03.011 20537501

[69] Fan J Meng Q Guo G Xie Y Li X Xiu Y Effects of glutamine added to enteral nutrition on Peyer’s patch apoptosis in severely burned mice. Burns (2010) 36(3):409–17. 10.1016/j.burns.2009.05.020 19783102

[70] Motta Neto R Guimaraes SB Silva SL Cruz JN Dias T Vasconcelos PR Glutamine or whey-protein supplementation on alloxan-induced diabetic rats. Effects on CD4+ and CD8+ lymphocytes. Acta Cir Bras (2007) 22(3):215–9. 10.1590/S0102-86502007000300010 17546295

[71] Chu CC Hou YC Pai MH Chao CJ Yeh SL. Pretreatment with alanyl-glutamine suppresses T-helper-cell-associated cytokine expression and reduces inflammatory responses in mice with acute DSS-induced colitis. J Nutr Biochem (2012) 23(9):1092–9. 10.1016/j.jnutbio.2011.06.002 22137260

[72] Hou YC Liu JJ Pai MH Tsou SS Yeh SL. Alanyl-glutamine administration suppresses Th17 and reduces inflammatory reaction in dextran sulfate sodium-induced acute colitis. Int Immunopharmacol (2013) 17(1):1–8. 10.1016/j.intimp.2013.05.004 23721689

[73] Hsiung YC Liu JJ Hou YC Yeh CL Yeh SL. Effects of dietary glutamine on the homeostasis of CD4+ T cells in mice with dextran sulfate sodium-induced acute colitis. PLoS One (2014) 9(1):e84410. 10.1371/journal.pone.0084410 24416230 PMC3887000

[74] Fukatsu K Kudsk KA Zarzaur BL Wu Y Hanna MK DeWitt RC. TPN decreases IL-4 and IL-10 mRNA expression in lipopolysaccharide stimulated intestinal lamina propria cells but glutamine supplementation preserves the expression. Shock (2001) 15(4):318–22. 10.1097/00024382-200115040-00012 11303733

[75] Ren W Li Y Yu X Luo W Liu G Shao H Glutamine modifies immune responses of mice infected with porcine circovirus type 2. Br J Nutr (2013) 110(6):1053–60. 10.1017/S0007114512006101 23351361

[76] Ren WK Liu SP Chen S Zhang FM Li NZ Yin J Dietary l-glutamine supplementation increases *Pasteurella multocida* burden and the expression of its major virulence factors in mice. Amino Acids (2013) 45(4):947–55. 10.1007/S00726-013-1551-8 23884693

[77] Engel JM Pitz S Muhling J Menges T Martens F Kwapisz M Role of glutamine administration on T-cell derived inflammatory response after cardiopulmonary bypass. Clin Nutr (2009) 28(1):15–20. 10.1016/j.clnu.2008.08.007 18835506

[78] Chen S Liu S Zhang F Ren W Li N Yin J Effects of dietary l-glutamine supplementation on specific and general defense responses in mice immunized with inactivated *Pasteurella multocida* vaccine. Amino Acids (2014) 46(10):2365–75. 10.1007/s00726-014-1789-9 24993936

[79] Le A Lane AN Hamaker M Bose S Gouw A Barbi J Glucose-independent glutamine metabolism via TCA cycling for proliferation and survival in B cells. Cell Metab (2012) 15(1):110–21. 10.1016/j.cmet.2011.12.009 22225880 PMC3345194

[80] Zhang G Ducatelle R Pasmans F D’Herde K Huang L Smet A Effects of *Helicobacter suis* gamma-glutamyl transpeptidase on lymphocytes: modulation by glutamine and glutathione supplementation and outer membrane vesicles as a putative delivery route of the enzyme. PLoS One (2013) 8(10):e77966 10.1371/journal.pone.0077966 24147103 PMC3797756

[81] Tian J Hao L Chandra P Jones DP Willams IR Gewirtz AT Dietary glutamine and oral antibiotics each improve indexes of gut barrier function in rat short bowel syndrome. Am J Physiol Gastrointest Liver Physiol (2009) 296(2):G348–55. 10.1152/ajpgi.90233.2008 19095767 PMC2643904

[82] Fan J Li G Wu L Tao S Wang W Sheng Z Parenteral glutamine supplementation in combination with enteral nutrition improves intestinal immunity in septic rats. Nutrition (2015) 31(5):766–74. 10.1016/j.nut.2014.11.021 25837225

[83] Iwata M Hirakiyama A Eshima Y Kagechika H Kato C Song SY. Retinoic acid imprints gut-homing specificity on T cells. Immunity (2004) 21(4):527–38. 10.1016/j.immuni.2004.08.011 15485630

[84] Mora JR von Andrian UH. Role of retinoic acid in the imprinting of gut-homing IgA-secreting cells. Semin Immunol (2009) 21(1):28–35. 10.1016/j.smim.2008.08.002 18804386 PMC2663412

[85] Kaetzel CS. Cooperativity among secretory IgA, the polymeric immunoglobulin receptor, and the gut microbiota promotes host-microbial mutualism. Immunol Lett (2014) 162(2 Pt A):10–21. 10.1016/j.imlet.2014.05.008 24877874 PMC4246051

[86] Cao AT Yao S Gong B Elson CO Cong Y. Th17 cells upregulate polymeric Ig receptor and intestinal IgA and contribute to intestinal homeostasis. J Immunol (2012) 189(9):4666–73. 10.4049/jimmunol.1200955 22993206 PMC3478497

[87] Ivanov II Littman DR. Segmented filamentous bacteria take the stage. Mucosal Immunol (2010) 3(3):209–12. 10.1038/mi.2010.3 20147894 PMC3010405

[88] Jiang HQ Bos NA Cebra JJ. Timing, localization, and persistence of colonization by segmented filamentous bacteria in the neonatal mouse gut depend on immune status of mothers and pups. Infect Immun (2001) 69(6):3611–7. 10.1128/IAI.69.6.3611-3617.2001 11349021 PMC98348

[89] Bruno ME Frantz AL Rogier EW Johansen FE Kaetzel CS Regulation of the polymeric immunoglobulin receptor by the classical and alternative NF-kappaB pathways in intestinal epithelial cells. Mucosal Immunol (2011) 4(4):468–78. 10.1038/mi.2011.8 21451502 PMC3125104

[90] Hooper LV Wong MH Thelin A Hansson L Falk PG Gordon JI. Molecular analysis of commensal host-microbial relationships in the intestine. Science (2001) 291(5505):881–4. 10.1126/science.291.5505.881 11157169

[91] Kvale D Brandtzaeg P. Constitutive and cytokine induced expression of HLA molecules, secretory component, and intercellular adhesion molecule-1 is modulated by butyrate in the colonic epithelial cell line HT-29. Gut (1995) 36(5):737–42. 10.1136/gut.36.5.737 7797124 PMC1382679

[92] Schneeman TA Bruno ME Schjerven H Johansen FE Chady L Kaetzel CS. Regulation of the polymeric Ig receptor by signaling through TLRs 3 and 4: linking innate and adaptive immune responses. J Immunol (2005) 175(1):376–84. 10.4049/jimmunol.175.1.376 15972671

[93] Sarkar J Gangopadhyay NN Moldoveanu Z Mestecky J Stephensen CB. Vitamin A is required for regulation of polymeric immunoglobulin receptor (pIgR) expression by interleukin-4 and interferon-gamma in a human intestinal epithelial cell line. J Nutr (1998) 128(7):1063–9. 9649586 10.1093/jn/128.7.1063

[94] Takenouchi-Ohkubo N Asano M Chihaya H Chung-Hsuing WU Ishikasa K Moro I. Retinoic acid enhances the gene expression of human polymeric immunoglobulin receptor (pIgR) by TNF-alpha. Clin Exp Immunol (2004) 135(3):448–54. 10.1111/j.1365-2249.2004.02398.x 15008977 PMC1808977

[95] Ren W Yin J Wu M Liu G Yang G Xion Y Serum amino acids profile and the beneficial effects of l-arginine or l-glutamine supplementation in dextran sulfate sodium colitis. PLoS One (2014) 9(2):e88335 10.1371/journal.pone.0088335 24505477 PMC3914992

[96] Ren WK Yin J Zhu XP Liu G Li NZ Peng YY Glutamine on intestinal inflammation: a mechanistic perspective. Eur J Inflammation (2013) 11(2):315–26.

[97] Hirota K Turner JE Villa M Duarte JH Demengeot J Steinmetz OM Plasticity of Th17 cells in Peyer’s patches is responsible for the induction of T cell-dependent IgA responses. Nat Immunol (2013) 14(4):372–9. 10.1038/ni.2552 23475182 PMC3672955

[98] Kruglov AA Grivennikov SI Kuprash DV Winsauer C Prepens S Seleznik GM Nonredundant function of soluble LTalpha3 produced by innate lymphoid cells in intestinal homeostasis. Science (2013) 342(6163):1243–6. 10.1126/science.1243364 24311691

[99] Arsenescu R Bruno ME Rogier EW Stefka AT McMahan AE Wright TB Signature biomarkers in Crohn’s disease: toward a molecular classification. Mucosal Immunol (2008) 1(5):399–411. 10.1038/mi.2008.32 19079204

[100] Frantz AL Bruno ME Rogier EW Tuna H Cohen DA Bondada S Multifactorial patterns of gene expression in colonic epithelial cells predict disease phenotypes in experimental colitis. Inflamm Bowel Dis (2012) 18(11):2138–48. 10.1002/ibd.22923 23070952 PMC3476470

[101] Smits HH Gloudemans AK van Nimwegen M Willart MA Soullie T Muskens F Cholera toxin B suppresses allergic inflammation through induction of secretory IgA. Mucosal Immunol (2009) 2(4):331–9. 10.1038/mi.2009.16 19404246

[102] Matysiak-Budnik T Moura IC Arcos-Fajardo M Lebreton C Menard S Candalh C Secretory IgA mediates retrotranscytosis of intact gliadin peptides via the transferrin receptor in celiac disease. J Exp Med (2008) 205(1):143–54. 10.1084/jem.20071204 18166587 PMC2234361

[103] Wu G Amino Acids: Biochemistry and Nutrition. Boca Raton, FL: CRC Press (2013).

[104] Li P Yin YL Li DF Kim SW Wu G Amino acids and immune function. Br J Nutr (2007) 98:237–52. 10.1027/S000711450769936X 17403271

[105] Hou YQ Yin YL Wu G Dietary essentiality of “nutritionally nonessential amino acids” for animals and humans. Exp Biol Med (2015) 240:997–1007. 10.1177/1535370215587913 PMC4935284 26041391

[106] Yi D Hou YQ Wang L Ouyang WJ Long MH Zhao D L-Glutamine enhances enterocyte growth via activation of the mTOR signaling pathway independently of AMPK. Amino Acids (2015) 47:65–78. 10.1007/s00726-014-1842-8 25280462

